# Verifiable Hypotheses for Thymosin β4-Dependent and -Independent Angiogenic Induction of *Trichinella spiralis*-Triggered Nurse Cell Formation

**DOI:** 10.3390/ijms141223492

**Published:** 2013-11-29

**Authors:** Mee Sun Ock, Hee-Jae Cha, Yung Hyun Choi

**Affiliations:** 1 Department of Parasitology and Genetics, Kosin University College of Medicine, Busan 602-702, Korea; E-Mail: sunnyock@kosin.ac.kr; 2 Institute for Medical Science, Kosin University College of Medicine, Busan 602-702, Korea; 3 Department of Biochemistry, College of Oriental Medicine, Anti-Aging Research Center, Dongeui University, Busan 614-052, Korea

**Keywords:** thymosin β4, *Trichinella spiralis*, nurse cells, angiogenesis, hypoxia

## Abstract

*Trichinella spiralis* has been reported to induce angiogenesis for nutrient supply and waste disposal by the induction of the angiogenic molecule vascular endothelial cell growth factor (VEGF) during nurse cell formation. However, the action mechanism to induce VEGF in nurse cells by *T. spiralis* is not known. Hypoxia in nurse cells was suggested as a possible mechanism; however, the presence of hypoxic conditions in infected muscle or nurse cells and whether hypoxia indeed induces the expression of VEGF and subsequent angiogenesis in the infected muscle are both a matter of debate. Our recent studies have shown that thymosin β4, a potent VEGF inducing protein, is expressed in the very early stages of *T. spiralis* muscle infection suggesting the induction of VEGF in early stage nurse cells. Nevertheless, we now show that hypoxic conditions were not detected in any nurse cell stage but were detected only in the accumulated inflammatory cells. These studies propose that induction of angiogenesis by VEGF in *T. spiralis*-infected nurse cells was mediated by thymosin β4 and is unrelated to hypoxic conditions.

## 1. Introduction

Parasites are living organisms that grow and reproduce by stealing host resources that progress into the detriment of the host organism. Although parasites harm their hosts, it is in the best interest of the parasite not to kill the host because the parasite relies on the host body functions, such as digestion or blood circulation, to survive. Thus, parasites have evolved in the direction of maximizing efficiency but not affecting their host systems. Parasites, especially helminthic parasites cause countless morbidity, but the mortality for the parasitic diseases is very low [1]. *Trichinella spiralis* is a relatively small nematode that causes infections in host skeletal muscle and modifies and utilizes the host system very efficiently. The life cycle of *T. spiralis* begins with the enteral phase of infection when a person or an animal eats contaminated meat containing first stage muscle larvae. Digestive juices including pepsin and hydrochloric acid dissolve the capsule-like cyst and release the larvae. The larvae then pass into the small intestine and mature to adults that mate to produce newborn larvae (immature L1). Newborn larvae are passed into the tissue and enter the lymphatic system and blood vessels for subsequent body-wide distribution. The larvae eventually extravasate into the muscle fibers, which initiate the muscle phase of infection. Once in the muscle fibers, the larvae encyst, undergo development, become infective within 15 days, and remain for months to years [2,3].

## 2. Nurse Cell Formation and Angiogenesis

After *T. spiralis* enters the muscle cell, a morphologically changed portion of infected myocytes are directed to develop into nurse cells that presumably function to nourish the parasite as well as to protect it from the host immune response. The nurse cell-parasite complex is surrounded by a collagen capsule consisting predominantly of two collagen types, IV and VI, both of which are synthesized by the nurse cell. The parasite begins to secrete proteins within the matrix of the infected host muscle cell 7 days after infection and the onset of host collagen type IV and type VI mRNA synthesis is also initiated between 7 and 8 days after infection. Collagen type IV synthesis then ceases on about 26 days after infection, while the expression of type VI collagen still remains throughout the infection at a low level [4,5].

In order to maintain a long-term host-parasite relationship, the parasite must remain metabolically active by maintaining nutrient acquisition and appropriate waste disposal. *T. spiralis* accomplishes these two tasks by attracting a highly permeable set of blood vessels to the surface of the outer collagen capsule. In this way, the larvae are assured a constant source of small molecular weight metabolites while also removing metabolic products from their environment. *T. spiralis* accomplishes this is by the initiation of angiogenesis.

Baruch and Despommier [6] conducted transcardial perfusion along with the injection of a plastic that polymerized *in situ* in *T. spiralis*-infected mice to find that simple, complex, and hypercomplex vascular networks were found only around infected myocytes. They suggested that the vascular retes are the result of *de novo* angiogenesis induced during infection and that the parasite may elicit angiogenesis directly through secretion of unique products or may elicit a change in the nurse cells that in turn results in the growth of new blood vessels. Capó *et al.* [7] first showed that vascular endothelial growth factor (VEGF), a potent angiogenic stimulator, was induced by *T. spiralis* infection. They found that both VEGF mRNA and VEGF peptide were detected in the developing nurse cell cytoplasm from day 7 up to 16 months after infection. In addition, VEGF was also detected in cells in the area immediately surrounding the nurse cells on days 15 and 17. They also proposed that hypoxia is induced by *T. spiralis* within the developing nurse cells some time prior to the up-regulation of VEGF, perhaps as early as day 7, and a constant state of hypoxia is maintained to explain the continued presence of VEGF in nurse cells [5,7]. However, no further studies were conducted to prove that the hypoxic condition actually occurs in the course of nurse cell formation. In addition, it is still unknown whether hypoxia stimulates the expression of VEGF mRNA and VEGF peptide in *T. spiralis*-infected nurse cells. Taken together, hypoxia seems to be the key clue to solving the action mechanism of angiogenic induction in *T. spiralis*-infected nurse cells.

## 3. Hypoxia and VEGF Induction in *T. spiralis*-Infected Nurse Cells

From the early stages of muscle infection, angiogenic induction in *T. spiralis*-infected nurse cells is mediated by elevated VEGF levels [7]. Therefore, identifying how VEGF is induced in *T. spiralis*-infected nurse cells can elucidate the action mechanism of angiogenic induction by parasite infection. Hypoxia is a major inducer of VEGF expression in almost all cell types examined. Transcriptional up-regulation of VEGF is mediated by the specific binding of hypoxia-inducible factor-1 (HIF-1) to the VEGF promoter hypoxic response element (HRE). The HIF-1 transcription factor is a heterodimer composed of HIF-1α and HIF-1β [8]. Whereas HIF-1β levels are constitutive, HIF-1α is degraded under normoxic conditions. The regulation of HIF-1α stability is a complex process. During oxygen-dependent activity, HIF-1α activity and stability are controlled differently on the basis of normoxic and hypoxic conditions.

Under normoxic conditions, HIF-1α activity is prevented through HIF hydroxylation and the von Hippel-Lindau tumor suppressor protein (pVHL) binds to hydroxylated HIF-1α. pVHL bound to HIF-1α generates a complex by the binding of elongin-C, elongin-B, cullin-2, and ring-box 1 (RBX1) of E3 ubiquitin ligase, which targets HIF-1α for ubiquitination and degradation by the 26S proteasome [9,10]. Under hypoxic conditions, HIF-1α hydroxylation is prevented and unhydroxylated HIF-1α cannot bind pVHL; therefore, pVHL accumulates in the cell. Accumulated HIF-1α translocates to the nucleus and heterodimerizes with HIF-1β to bind to HRE regions on nuclear DNA to promote the transcription of target genes involved in cell growth, angiogenesis, glucose metabolism, pH regulation, and cell survival/apoptosis [9,10]. During oxygen-independent activity, pVHL-independent HIF degradation occurs. HIF-1α degradation is mainly regulated by heat shock protein 90 (HSP90), which protects proteins from misfolding and degradation through its ATPase activity. The receptor for activated C-kinase 1 (RACK1), a novel HIF-1α-interacting protein, can promote proteasome-dependent degradation of HIF-1α in an oxygen-independent manner and competes with HSP90 to bind to the PAS-A domain of HIF-1α [9,10]. HSP90 binding stabilizes HIF-1α by excluding RACK1, which binds elongin-C and recruits elongin-B and other components of E3 ubiquitin ligase to HIF-1α [9,10]. Even though hypoxia is the major stimulator of VEGF expression and angiogenesis, hypoxia independent regulation of VEGF also exists via the modulation of HIF-1α stability in an oxygen independent manner or HIF independent regulation of VEGF expression [10].

## 4. Thymosin β4 and *T. spiralis* Infection

We previously reported that thymosin β4, a major factor that induces VEGF, showed increased expression in muscle fibers 10 days after *T. spiralis* infection at both mRNA and protein level, and VEGF expression remained high in nurse cells for 6 weeks when the formation of the nurse cell complex was completed [11,12]. Thymosin β4 is an actin sequestering protein that has been reported to be a multi-functional protein with various biological roles, such as promoting angiogenesis [13–15], tumor metastasis [12,16–19], wound healing [20,21], hair growth [22,23], anti-apoptotic [24,25], and anti-inflammatory [26,27] effects. Our previous study showed that VEGF was up-regulated in thymosin β4 over-expressing B16-F10 lung tumor cells [13]. Conversely, down-regulation of VEGF was observed *in situ* in thymosin β4 knockdown hearts, suggesting that appropriate VEGF expression may require thymosin β4 [28]. Furthermore, we also found that thymosin β4 induces the expression of VEGF under normoxic conditions by increasing HIF-1α protein stability [29]. Therefore, increased levels of thymosin β4 in *T. spiralis*-infected nurse cells may increase VEGF levels to stimulate angiogenesis.

## 5. Hypoxia, HIF-1α, and VEGF in *T. spiralis*-Infected Nurse Cells

Our previous report also showed that hypoxic conditions are not present in *T. spiralis*-infected nurse cells. We used piminodazole hydrochloride, a chemical detector of hypoxic place, to find hypoxic condition in infected nurse cells. As the results of immunofluorescence with dual primary antibodies of anti-piminodazole hydrochloride and anti-HIF-1α, hypoxic conditions were not detected in nurse cells but were detected in accumulated lymphocytes surrounding nurse cells, particularly in degenerating nurse cells [11]. HIF-1α expression patterns were co-localized with hypoxic places suggesting hypoxia does not occur in *T. spiralis*-infected nurse cells but is present in the crowd of lymphocytes. The HIF-1α protein levels were dramatically increased from 21 days after infection until 42 days after infection, also suggesting the hypoxia is not related VEGF induction in nurse cells [11]. VEGF protein was also detected in the crowd of lymphocytes and the elevation of VEGF in late infection stages may be caused by hypoxic induction in the crowd of lymphocytes. Other growth factors including fibroblast growth factor (FGF)-1, FGF-2, epidermal growth factor (EGF), and insulin-like growth factor (IGF-1), which stimulate angiogenesis, were also checked. Only FGF-1 was increased in early stage infection and other growth factors such as FGF-2 and IGF-1 were highly induced from 14 days after infection. The expression of EGF did not change in any stage of *T. spiralis* infection. These data suggest that FGF-1 could be involved in angiogenic stimulation during nurse cell formation and FGF-2 and IGF-1 may be related with inflammation by lymphocyte accumulation [11].

## 6. Conclusions and Future Studies

**Figure 1.**
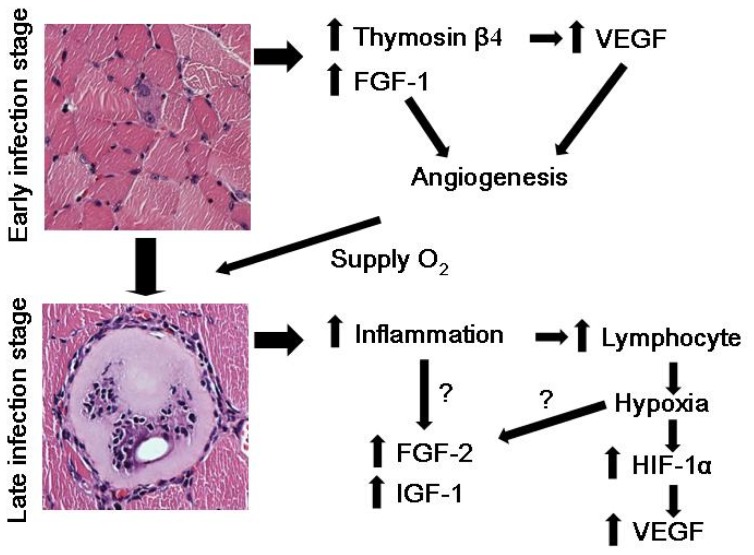
Proposed mechanism of thymosin β4-mediated vascular endothelial cell growth factor (VEGF) induction in *T. spiralis*-infected nurse cells. Induction of thymosin β4 in early infection stimulates expression of VEGF to induce angiogenesis in early stage nurse cells. Induction of fibroblast growth factor (FGF)-1 also stimulates angiogenesis and the induction of angiogenesis can supply oxygen to nurse cells without having to go through a hypoxic state. At late stages of infection, inflammation around infected nurse cells occurs and lymphocytes accumulate around damaged nurse cells. The hypoxic conditions due to the accumulation of lymphocytes protect hypoxia-inducible factor (HIF)-1α from hydroxylation and degradation resulting in the transcriptional activation of VEGF expression. Other factors including FGF-2 and insulin-like growth factor (IGF)-1 may also be involved in inflammation or the hypoxic stimulation of gene expression.

According to these data, we suggest that the induction of thymosin β4 in early stages of infection stimulates expression of VEGF to induce angiogenesis in early stage nurse cells. Induction of FGF-1also stimulates angiogenesis and the induction of angiogenesis can supply oxygen to nurse cells through a mechanism other than the hypoxic state (Figure 1). At late stages of infection, inflammation around infected nurse cells occurs and lymphocytes accumulate around the damaged nurse cells. The hypoxic condition, due to the accumulation of lymphocytes, protects HIF-1α from hydroxylation and degradation resulting in the transcriptional activation of VEGF expression. Other factors including FGF-2 and IGF-1 may also be involved in inflammation or the hypoxic stimulation of gene expression (Figure 1). However, several unsolved questions still remain. First, there is no direct evidence demonstrating that thymosin β4 actually mediates the angiogenic stimulation in nurse cell formation. Direct induction of VEGF by thymosin β4 in nurse cell formation should be confirmed. In addition, the effect of thymosin β4 over-expression or knock down in animal model on VEGF expression and angiogenesis in nurse cell formation should be examined. These studies can probe the direct involvement of thymosin β4 in VEGF expression and angiogenesis during nurse cell formation. Furthermore, how *T. spiralis* infection can stimulate thymosin β4 in nurse cells should also be studied to elucidate the action mechanism of thymosin β4 induction by parasite infection and also provide advanced knowledge about *T. spiralis* infection.
